# Analysis of the complement and molecular evolution of tRNA genes in cow

**DOI:** 10.1186/1471-2164-10-188

**Published:** 2009-04-24

**Authors:** Dave TP Tang, Evgeny A Glazov, Sean M McWilliam, Wesley C Barris, Brian P Dalrymple

**Affiliations:** 1 CSIRO Livestock Industries, Queensland Biosciences Precinct, 306 Carmody Road, St Lucia, Qld 4067, Australia

## Abstract

**Background:**

Detailed information regarding the number and organization of transfer RNA (tRNA) genes at the genome level is becoming readily available with the increase of DNA sequencing of whole genomes. However the identification of functional tRNA genes is challenging for species that have large numbers of repetitive elements containing tRNA derived sequences, such as *Bos taurus*. Reliable identification and annotation of entire sets of tRNA genes allows the evolution of tRNA genes to be understood on a genomic scale.

**Results:**

In this study, we explored the *B. taurus *genome using bioinformatics and comparative genomics approaches to catalogue and analyze cow tRNA genes. The initial analysis of the cow genome using tRNAscan-SE identified 31,868 putative tRNA genes and 189,183 pseudogenes, where 28,830 of the 31,868 predicted tRNA genes were classified as repetitive elements by the RepeatMasker program. We then used comparative genomics to further discriminate between functional tRNA genes and tRNA-derived sequences for the remaining set of 3,038 putative tRNA genes. For our analysis, we used the human, chimpanzee, mouse, rat, horse, dog, chicken and fugu genomes to predict that the number of active tRNA genes in cow lies in the vicinity of 439. Of this set, 150 tRNA genes were 100% identical in their sequences across all nine vertebrate genomes studied. Using clustering analyses, we identified a new tRNA-Gly^CCC ^subfamily present in all analyzed mammalian genomes. We suggest that this subfamily originated from an ancestral tRNA-Gly^GCC ^gene via a point mutation prior to the radiation of the mammalian lineages. Lastly, in a separate analysis we created phylogenetic profiles for each putative cow tRNA gene using a representative set of genomes to gain an overview of common evolutionary histories of tRNA genes.

**Conclusion:**

The use of a combination of bioinformatics and comparative genomics approaches has allowed the confident identification of a set of cow tRNA genes that will facilitate further studies in understanding the molecular evolution of cow tRNA genes.

## Background

Transfer RNAs (tRNAs) are members of a family of ubiquitous molecules that provide the essential link between the genetic code and amino acids. tRNAs are a central component of ribosomal protein biosynthesis that acts by decoding template messenger RNA (mRNA) into the amino acid sequence of the encoded protein. Although the mechanistic role of tRNA in protein synthesis is well understood, evidence illustrating the complexity of evolution and expression of tRNA genes continues to emerge [1-3].

The tRNA multigene family usually comprises 20 amino acid accepting groups; each group may contain one or more tRNA members that recognize different codons commonly referred to as tRNA isoacceptors. Generally, the number of tRNA isoacceptors is highly variable amongst different genomes [4]. While variations in tRNA isoacceptor numbers is positively correlated to codon usage in bacteria and yeast [5], such correlation is absent in more advanced eukaryotic genomes such as frogs and humans [6]. tRNA isoacceptors usually exhibit close sequence similarity as they conserve the same sequence and structural elements used for identification by the same class of aminoacyl-tRNA synthetases [7]. However, in some cases tRNAs that charge different amino acids share higher sequence similarity than respective tRNAs in the same isoaccepting group [8]. This interesting observation may be explained by a phenomenon called "tRNA gene recruitment" where a tRNA from one amino acid family can be recruited to another family via a single point mutation [9]. These tRNA gene recruitment events complicate the evolutionary history of tRNA genes. However, a phylogenetic approach utilizing complete sets of tRNA genes from different genomes will allow us to trace a more complete history of tRNA genes and gain insight into the evolutionary processes that have shaped modern tRNA repertoires.

With the advent of multiple genome sequencing projects, entire tRNA repertoires have been identified in many genomes. While features of tRNA genes are well defined, predicting functional tRNA genes in eukaryotic genomes can be difficult. Sequencing of vertebrate genomes revealed that tRNA-derived sequences are often contained within retrotransposon insertions that give rise to different families of short interspersed elements (SINEs) [10]. In the genomes of animals, the number of SINEs present often exceeds 10^4 ^[11]. For example, approximately 1.6% of the *Bos taurus *genome is predicted to comprise of SINEs containing tRNA-derived sequences [12]. These complications present a serious challenge for the accurate annotation of biochemically active tRNA genes as SINEs often retain many sequence and structural features of the authentic tRNA genes [10,13].

Identification of functional non-coding RNAs in genomic sequences depends heavily on the analysis of conserved RNA secondary structure in order to make accurate predictions [14]. The most commonly used program for making tRNA predictions is tRNAscan-SE [15], a program that analyzes intrinsic features of known functional tRNA genes including the internal Pol III promoter sites and conserved tRNA secondary structure. However, tRNAscan-SE produces large numbers of false positives in genomes where tRNA related sequences and SINEs are common [16,17]. For the prediction and annotation of mouse tRNA genes, the strategy to counter this problem was to identify SINEs that have been predicted as tRNA genes and use comparative genomics to identify functional tRNA genes [16]. In that study, SINEs were identified using RepeatMasker [18], a program that identifies putative repeat elements in genomic sequences by comparison to a pre-compiled database of repetitive elements. Although RepeatMasker can effectively identify many genomic repeat sequences, it is unable to identify novel lineage-specific SINEs. To overcome this, a comparative genomics approach using the human genome was used to identify sets of orthologous tRNA genes that are probably functional. This strategy allowed the refining of a large set of putative mouse tRNA genes (2,764) to a smaller set of possibly functional mouse tRNA genes (335) [16].

For our analysis of the *B. taurus *genome, we were challenged by a large number of tRNA-like sequences due to SINE activity. In order to provide a comprehensive annotation of functional tRNA genes in the *B. taurus *genome we used a combination of approaches which involved tRNAscan-SE, RepeatMasker and comparative genomics to overcome the issue of SINEs. We then conducted cluster analyses on our set of putative tRNA genes, to gain insight into the evolutionary histories of cow tRNA genes. This in turn will help further the understanding of tRNA gene evolution.

## Results

### Identification of cow tRNA genes

The initial analysis of the cow genome using tRNAscan-SE identified 191,073 and 29,978 tRNA-like sequences positioned evenly across assembled cow chromosomes and unordered genomic contigs respectively (Table 1, Additional file 1). Of these sequences, 163,490 and 25,693 on the assembled chromosomes and unordered contigs respectively were classified as pseudogenes by tRNAscan-SE. Together this is by far the largest reported number of tRNA-like sequences amongst vertebrate genomes, demonstrating the extent of amplification of tRNA-derived sequences in the cow. In comparison similar analyses performed in other mammals identified a total of 2,764 tRNA genes and 22,314 pseudogenes in the mouse genome [16], a total of 175,943 tRNA genes and pseudogenes in the rat genome [17] and 497 tRNA genes and 324 tRNA pseudogenes in the human genome [19]. All these data illustrate a great variability in tRNA-derived sequence copy numbers owing mainly to the activity of SINEs [16,17,19]. Given the large numbers of tRNA-like sequences identified in our initial analysis, we reasoned that the remaining sets of 27,583 and 4,285 putative tRNA genes on assembled chromosomes and unordered contigs respectively are likely to contain SINEs and tRNA-related sequences that were misclassified as tRNA genes by tRNAscan-SE. To address this problem, we used RepeatMasker to identity repetitive elements.

**Table 1:** Summary of tRNA gene predictions in the cow genome.

Amino acid/Pseudo genes	Numbers of tRNAscan-SE predictions		Numbers of RepMask^3 ^tRNAs		RepMask % of removed tRNAs		Numbers of tRNAs without RE^4^		Numbers of tRNAs with 95% similarity	
	
	Chr1-X^1^	ChrUn^2^	Chr 1-X	ChrUn	Chr 1-X	ChrUn	Chr 1-X	ChrUn	Chr 1-X	ChrUn
Ala	293	41	216	30	73.7	73.2	77	11	18	2
Arg	811	120	755	109	93.1	90.8	56	11	18	5
Asn	57	24	25	4	43.9	16.7	32	20	20	11
Asp	316	44	238	29	75.3	65.9	78	15	12	5
Cys	12062	2061	12033	2059	99.8	99.9	29	2	25	0
Gln	142	30	79	15	55.6	50.0	63	15	14	9
Glu	2163	330	817	113	37.8	34.2	1346	217	14	10
Gly	7449	927	7141	881	95.9	95.0	308	46	22	10
His	193	44	178	36	92.2	81.8	15	8	10	8
Ile	59	9	29	5	49.2	55.6	30	4	17	3
Leu	144	30	92	11	63.9	36.7	52	19	12	4
Lys	300	57	93	20	31.0	35.1	207	37	14	7
Met	39	7	10	2	25.6	28.6	29	5	12	2
Phe	352	61	324	58	92.1	95.1	28	3	11	3
Pro	27	10	8	1	29.6	10.0	19	9	15	9
Ser	539	98	491	88	91.1	89.8	48	10	14	2
Thr	48	7	14	5	29.2	71.4	34	2	8	0
Trp	1558	191	1519	191	97.5	100.0	39	0	5	0
Tyr	822	145	799	144	97.2	99.3	23	1	15	0
Val	209	49	148	20	70.8	40.8	61	29	22	17
Pseudogenes	163490	25693	158551	25038	97.0	97.5	-	-		

Total	191073	29978	183560	28859	96.1	96.3	2574	464	298	107

^1^Chr1-X refers to assembled cow chromosomes

^2^ChrUn refers to the unordered cow contigs

^3^RepMask refers to the RepeatMasker program

^4^RE refers to repetitive elements.

### Identification of repetitive elements

Following the tRNAscan-SE analysis, RepeatMasker identified the majority of tRNA gene predictions to be repetitive elements (Table 1). Of the total 31,868 putative tRNA genes for the 20 standard amino acid families on both assembled chromosomes and unordered genomic contigs, RepeatMasker annotated 28,830 as repetitive elements, removing 90.5% of the putative tRNA genes (Table 1). Similarly, of the total 221,051 predicted tRNA genes on both assembled chromosomes and unordered genomic contigs, RepeatMasker annotated 212,419 as repetitive elements corresponding to 96.1% of the predictions (Table 1). After the initial RepeatMasker filter, tRNA genes in the glutamic acid, glycine and lysine amino acid families were still represented in large numbers with 1346, 308 and 207 copies respectively (Table 1). Similar observations of disproportionate increases in tRNA gene copy numbers of certain tRNA families can also be observed in the dog genome . We used a comparative genomics approach to further distinguish between functional tRNA genes and tRNA-related sequences.

### Defining a set of highly conserved vertebrate tRNA genes

Sequences with functional activity are subject to selective pressures that prevent the fixation of mutations that would compromise functionality. In contrast, most repetitive elements have lost their functional activity and evolve neutrally by accumulating random mutations, which results in much weaker evolutionary conservation in these sequences [20]. By comparing tRNA gene sets of cow, horse, dog, human, chimpanzee, mouse, rat, chicken and fugu, we found many highly conserved tRNA genes despite the evolutionary distances between the organisms. We found a unique set of 22 putative tRNA gene sequences that were completely identical in all of the genomes analyzed. Many of these 22 unique tRNA sequences were present in more than one copy in cow. In total, this set consisted of a set of 150 tRNA genes from 16 amino acid families.

**Figure 1:**
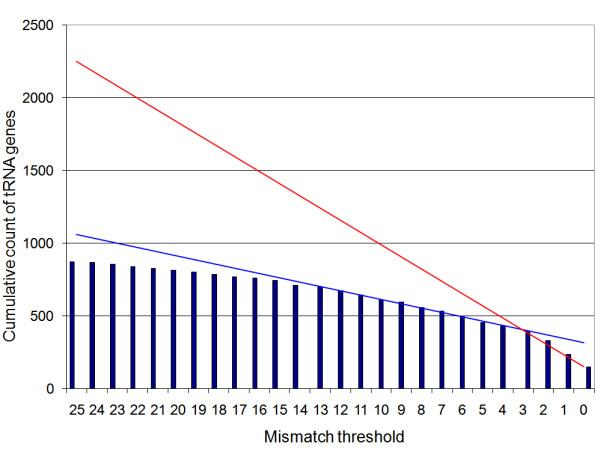
**Summary of the extent of cow tRNA gene sequence variability**. Each cow tRNA gene is compared with tRNA genes from the horse, dog, mouse, rat, human, chimpanzee, chicken and fugu genomes. The unit of mismatch is in single nucleotide bases and represents a threshold of similarity. For example a mismatch threshold of 3 includes cow tRNA genes that have 3 or less base differences to tRNA genes from other genomes. Trend lines for counts for 0–3 mismatches are shown in red and for counts for 4–13 mismatches are shown in blue.

**Figure 2:**
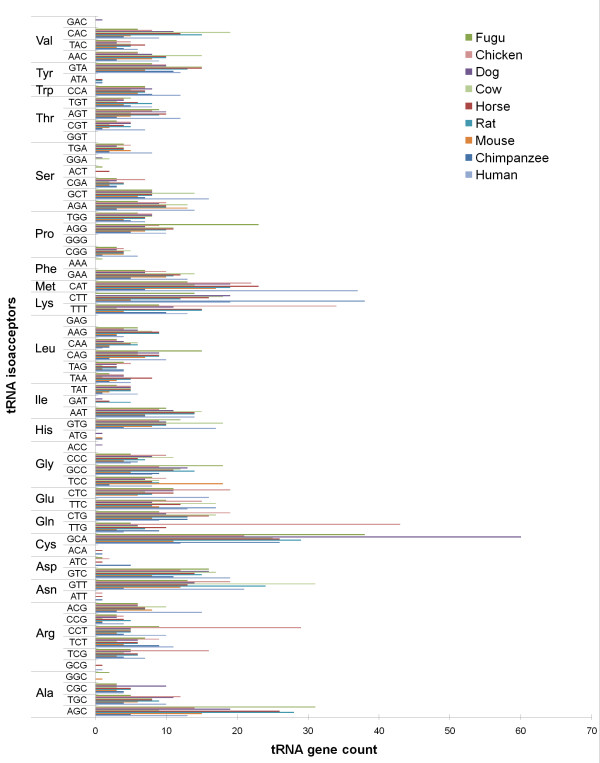
**The distribution of tRNA genes of vertebrate genomes grouped by amino acid specificity and by the anticodon**. Dog tRNA-Lys^CTT ^and Horse tRNA-Glu^CTC ^were not plotted in this graph as many of these predictions may have contained tRNA-like sequences present in repetitive elements.

Most other cow tRNA genes have accumulated at least one point mutation during the evolution of the vertebrates (Figure 1), since a tRNA gene may accumulate mutations while still retaining biological function. The 95% sequence similarity threshold used to identify a true set of cow tRNA genes corresponds to approximately 3 mismatches; this threshold also coincides with the end of the linear growth in cumulative tRNA counts on the plot (Figure 1). Application of the 95% threshold value resulted in the identification of the 403 different genes, represented by 133 unique sequences that contain 41 tRNA isoacceptors (Table 2), capable of decoding 55 of the 61 sense codons (Additional file 2) according to the wobble rules proposed by Gutherie and Abelson [21], where the codon's third position U and C are generally decoded by a single tRNA species. To identify tRNAs decoding the 6 codons not included in the set of tRNAs identified using the > = 95% sequence identity threshold, elongator methionine tRNA(s) and tRNA(s) directing the insertion of selenocysteine (Sec) we manually curated the relevant subsets of the 3,038 putative tRNA genes. We found a further 23 unique tRNA sequences encoded by 36 genes (Additional file 1). As a result, we provide a set of 439 cow tRNA genes represented by 156 unique tRNA genes capable of decoding all of the 61 sense codons (Additional file 3). Overall, the distribution of these 439 cow tRNA genes across the anticodons follows a similar trend as in the other vertebrate genomes (Figure 2).

**Table 2:** Summary of cow tRNA genes identified at the 95% similarity threshold.

Isoaccepting families		tRNA isoacceptor genes		Unique tRNAs^1^
Amino acid	number	anticodon	number	

Ala	20	AlaAGC	9	5
		AlaCGC	3	1
		AlaTGC	8	5

Arg	23	ArgACG	10	2
		ArgCCG	1	1
		ArgCCT	5	4
		ArgTCG	4	3
		ArgTCT	3	3

Asn	31	AsnGTT	31	9

Asp	17	AspGTC	17	5

Cys	24	CysGCA	24	10

Gln	23	GlnCTG	17	5

		GlnTTG	6	4

Glu	24	GluCTC	7	2
		GluTTC	17	6

Gly	32	GlyCCC	11	5
		GlyGCC	12	2
		GlyTCC	9	2

His	18	HisGTG	18	1

Ile	20	IleAAT	15	3
		IleTAT	5	2

Leu	16	LeuAAG	8	2
		LeuCAG	6	3
		LeuTAA	1	1
		LeuTAG	1	1

Lys	20	LysCTT	18	3
		LysTTT	2	1

Met	14	MetCAT	14	3

Phe	14	PheGAA	14	3

Pro	24	ProAGG	11	1
		ProCGG	5	1
		ProTGG	8	3

Ser	16	SerCGA	2	2
		SerGCT	14	7

Thr	8	ThrAGT	5	2
		ThrTGT	3	1

Trp	5	TrpCCA	5	1

Tyr	15	TyrGTA	15	6

Val	39	ValAAC	15	5
		ValCAC	19	3
		ValTAC	5	4

^1^Unique tRNAs are tRNA genes with at least 1 nucleotide difference from other genes with the same anticodon.

### Cluster analysis of 158 unique tRNA genes

**Figure 3:**
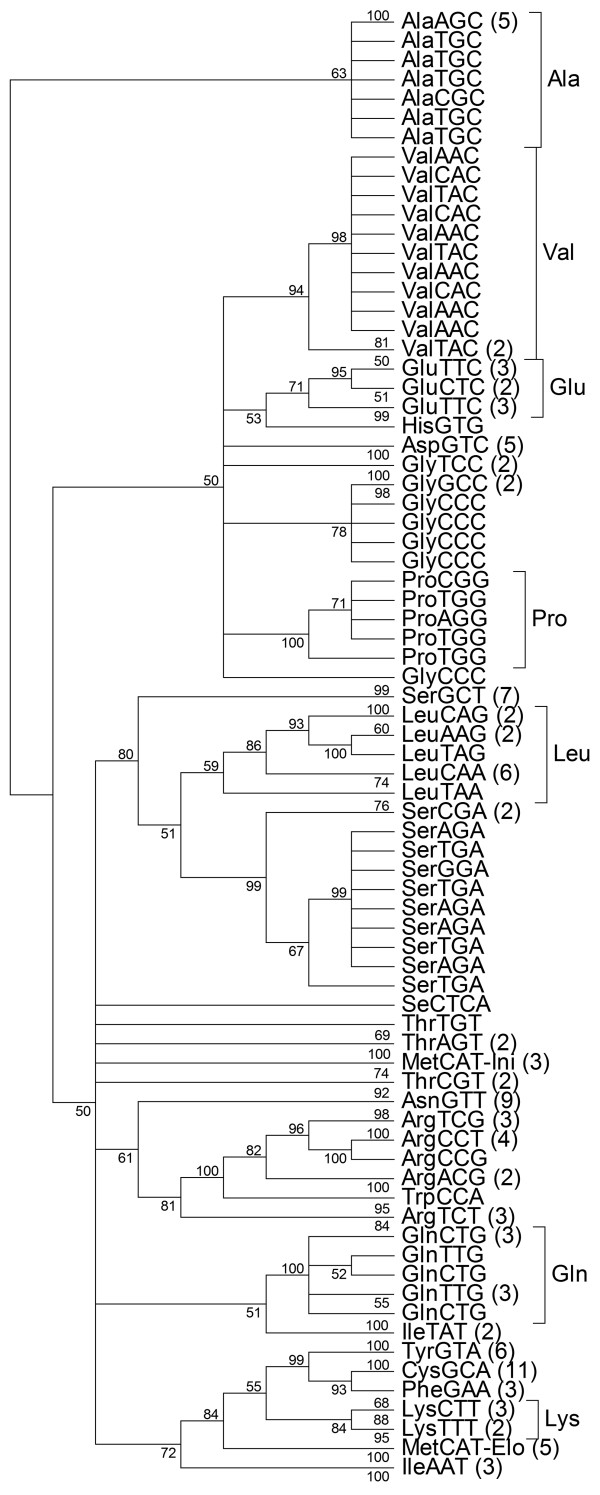
**Unrooted tree of 158 clustered unique cow tRNA gene sequences (out of a total of 441) inferred using Bayesian phylogenetic analysis**. The likelihood calculations were performed using General Time Reversible model. Posterior probabilities (PP) values are associated with each grouping of taxa and shown at each respective node.

**Figure 4:**
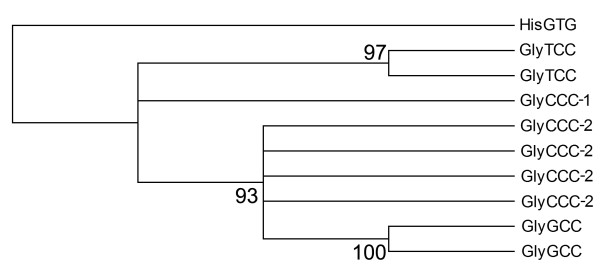
**A rooted phylogenetic tree constructed using Bayesian inference showing the evolutionary relationships between nine unique glycine tRNA gene sequences identified in the cow genome**. Two subfamilies of glycine tRNA genes with a CCC anticodon (GlyCCC-1 and GlyCCC-2) are present.

**Figure 5:**
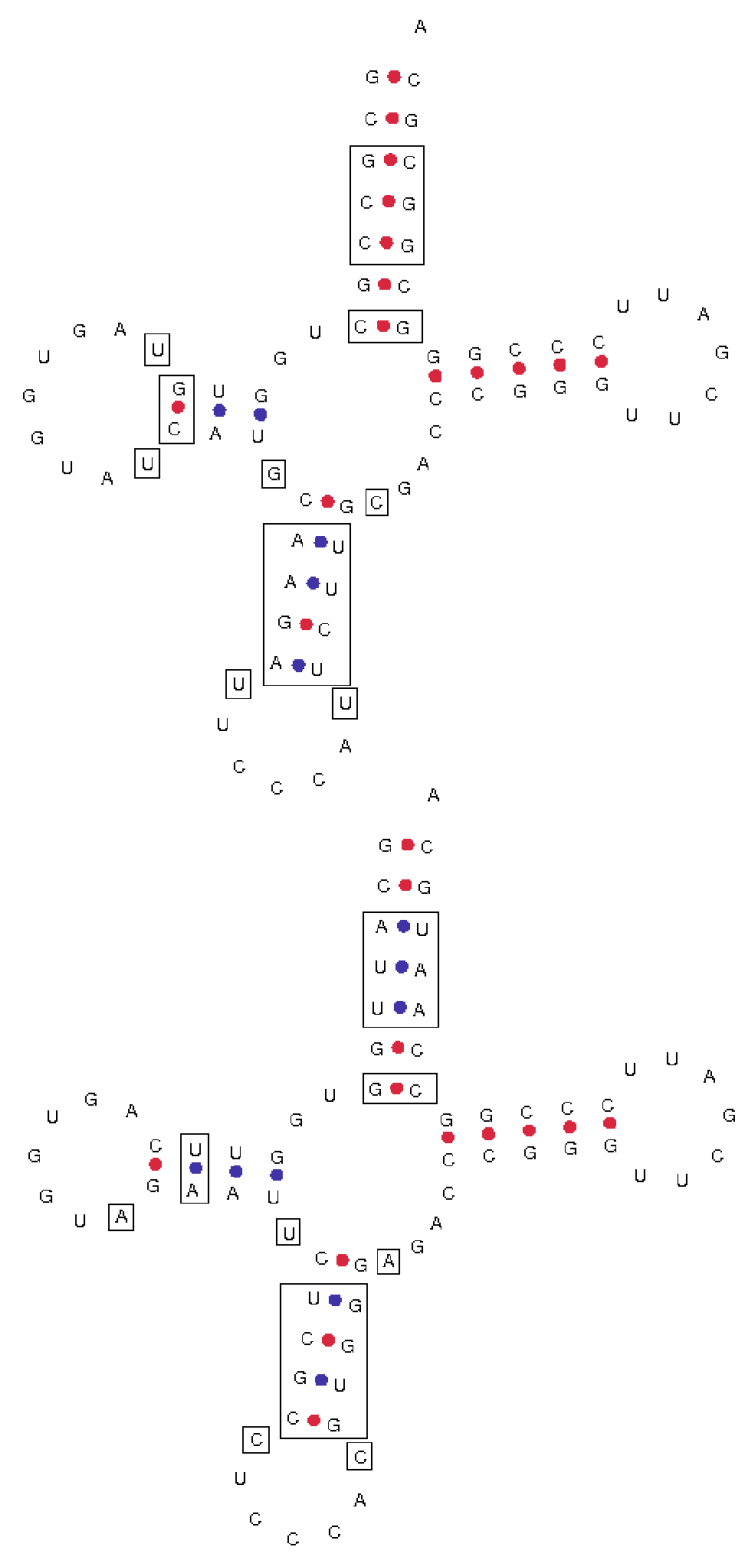
**Two tRNA-Gly^CCC ^genes displayed in the canonical tRNA cloverleaf secondary structure**. The upper and lower structures correspond to GlyCCC-1 and GlyCCC-2 respectively. Differences in nucleotides between the two tRNA molecules are highlighted with rectangles. Prediction of the secondary structure was done using tools available Genomic tRNA database website [26].

The set of 158 unique tRNA gene sequences was examined using cluster analysis. tRNA genes that carry the same amino acid generally formed clusters with ≥ 0.95 posterior probability (PP) (Figure 3). This observation concurs with the general consensus suggesting that tRNA genes that belong to the same amino acid family have evolved from a common tRNA. However not all tRNA genes in the same amino acid family formed clusters with a high PP value. tRNA genes from the serine, glutamatic acid, glycine, initiator methionine, threonine and arginine tRNA families could not be resolved to a single cluster. Interestingly, the tRNA gene sequence for tryptophan lay within a family of arginine specific tRNA genes, supported by a PP value of 1. While not all tRNA genes from the same amino acid family formed single clusters, most tRNA genes with the same anticodon clustered together. There were three exceptions; SerTGA, ValTAC and GlyCCC tRNA genes (Figure 3, Additional file 4, Additional file 5). This observation suggests that these tRNA genes may have different evolutionary histories. The multiple sequence alignment of glycine tRNA genes showed that the BoxB site is identical in all glycine tRNA genes, point mutations were observed at other sites (Additional file 6). A cluster analysis of unique tRNA-Gly genes revealed two distinct tRNA-Gly^CCC ^subfamilies (Figure 4). The first subfamily of tRNA-Gly^CCC ^differs from the second subfamily of tRNA-Gly^CCC ^that is closely related to the tRNA-Gly^GCC ^genes. Predicted secondary structures of these two tRNA-Gly^CCC ^genes illustrate various differences throughout the structure (Figure 5). We analyzed the distribution of these two families of tRNA-Gly^CCC ^genes among different genomes and find that the subfamily 1 tRNA-Gly^CCC ^genes are highly conserved among human, chimpanzee, mouse, rat, horse, dog, opossum, chicken, and fugu and usually present in two gene copies. However, only one member of this subfamily was observed in cow and no members of this subfamily were identified in the current assembly of the platypus genome (data not shown). In contrast, subfamily 2 tRNA-Gly^CCC ^genes appear to be only present in the mammals as no orthologs were detectable in the current assemblies of the chicken, lizard and fugu genomes.

### Phylogenetic profiles of putative cow tRNA genes

**Figure 6:**
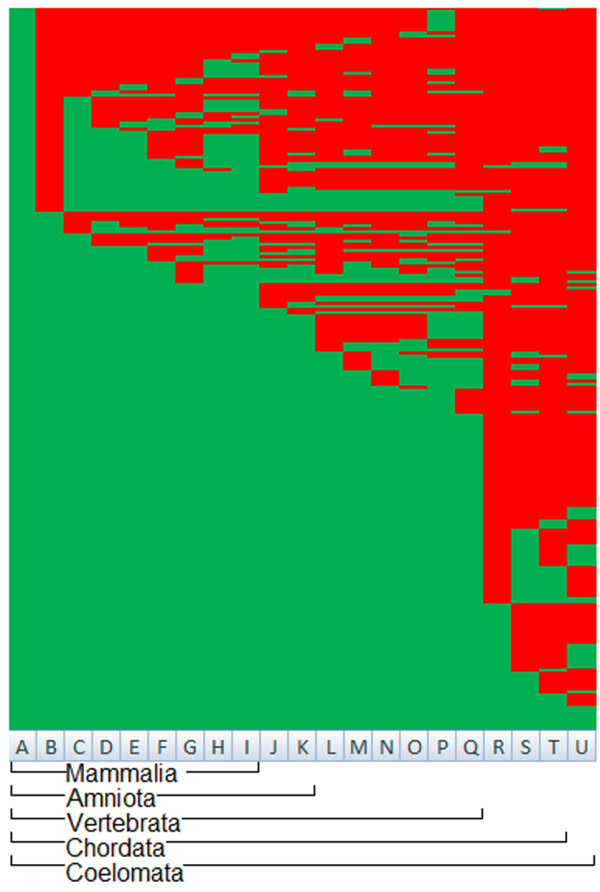
**Phylogenetic profiles of 232 unique putative cow tRNA genes of a total of 533**. Each row represents a positive BLAST hit of a putative cow tRNA gene to the tRNA from a query set. The rows are arranged in the same order of the amino acid tRNA families and anticodons as shown in figure 2. Each column represents a different genome where A – cow, B – horse, C – dog, D – mouse, E – rat, F – opossum, G – platypus, H – human, I – chimpanzee, J – chicken, K – green anole lizard, L – medaka, M – stickleback, N – fugu, O – tetraodon, P – zebrafish, Q – lamprey, R – sea squirt, S – lancelet, T – sea urchin and U – honey bee.

We created phylogenetic profiles of cow tRNA genes to identify the presence or absence of tRNA gene homologs in other genomes. We compared the cow tRNA gene repertoire to a representative set of genomes from mammals (horse, dog, mouse, rat, opossum, platypus, human and chimpanzee), fish (medaka, stickleback, fugu, tetraodon, zebrafish, and lamprey), reptile (green anole lizard), bird (chicken) and invertebrates (purple sea urchin, honey bee, lamprey, and lancelet). To be comprehensive we used the set of 31,868 cow putative tRNA genes identified using tRNAscan-SE and another 328,857 putative tRNA genes from the other 20 genomes (Additional file 7). Of the total cow tRNA genes, 533 tRNA genes (232 unique sequences) had at least one match at ≥ 95% outside the cow genome (Figure 6). The most common phylogenetic profile identifies cow tRNA gene orthologs only among vertebrate genomes (Additional file 8).

Additionally, more cow tRNA orthologs are identified among genomes that are less phylogenetically diverged. Using ≥ 95% sequence similarity threshold we identified, between 446 and 494 tRNA genes among mammalian genomes, 435 and 445 tRNA genes in chicken and lizard respectively and a range of 405 – 449 tRNA genes among fishes (Additional file 8) comparing the total of 31,868 putative cow tRNA genes. In contrast, only 103 – 155 cow tRNA genes were identified among invertebrate genomes using the same threshold (Additional file 8).

### Distribution of tRNA genes in the cow genome

**Figure 7:**
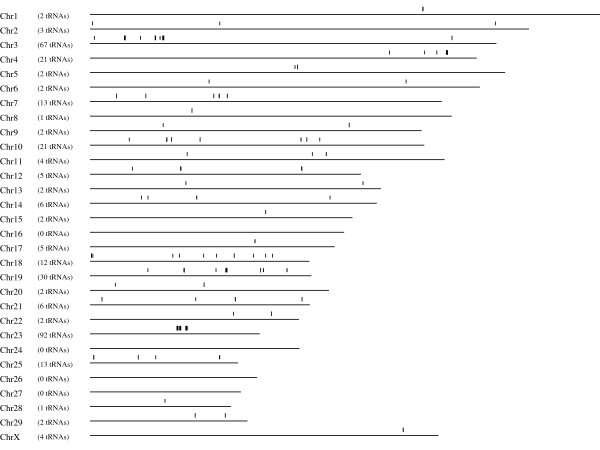
**The distribution of annotated tRNA genes in the cow genome**. Each vertical bar represents a single gene. The number of tRNA genes assigned to each chromosome is indicated as in some regions the genes are located too close together to be displayed as separate lines. 117 tRNA genes were located in unassigned segments of the cow genome.

tRNA genes generally occur in clusters, some of which contain large numbers of genes. The cow genome contains a number of such large clusters, in particular on chromosomes 3, 12, 19 and 23 (Figure 7). The organisation of the genes in these clusters is generally very similar to the equivalent clusters in the human genome (data not shown).

## Discussion

We performed a genome-wide analysis of the tRNA gene repertoire of the *Bos taurus *genome. Although our analyses were complicated by the wide spread distribution of SINEs, we were able to identify a representative set of tRNA genes in the cow. The impact of SINEs on tRNA gene prediction has been previously observed in other genomes. For example, the mouse genome contains over 25,000 tRNA genes and pseudogenes [16], while the rat genome contains over 175,000 tRNA genes and pseudogenes [17]. In dog, 401 tRNA-Lys^CTT ^genes were predicted, many of which are false positives due to a family of SINEs specific among carnivore genomes that have evolved from a tRNA-Lys^CTT ^[22].

For the accurate identification of tRNA genes in the mouse genome, putative mouse tRNA genes predicted by tRNAscan-SE were analyzed using RepeatMasker to remove tRNA-related SINEs and then cross-validated by identifying orthologous mouse-human tRNA genes [16]. We adopted a similar strategy for the annotation of cow tRNA genes except that we compared our putative tRNA genes to a larger set of genomes. While our RepeatMasker analysis was able to filter out a large number of false positives (~96% of total tRNA gene predictions), the remaining set of tRNA genes was still relatively large when compared to numbers observed in other vertebrate genomes. The majority of putative tRNA genes not identified as repetitive elements were derived from three amino acid families, glutamic acid, glycine and lysine suggesting that these might originate from recent expansion of ruminant-specific tRNA-derived repeat families. In fact the family of tRNA-derived SINEs present among ruminants that was created from a tRNA-Glu [23], may account for many mis-annotated tRNA genes in the glutamic acid family. These SINEs have maintained a high level of sequence similarity to authentic tRNA genes and have tRNA-like predicted secondary structures (Additional file 9). Elevated numbers in the glycine and lysine amino acid families are probably due to point mutations in the anticodon region of the tRNA-related sequence of this tRNA-Glu SINE, leading to TCC, CCC, CTT and TTT anticodons, which belong to the glycine and lysine tRNA gene families.

To help distinguish between well conserved tRNA-like sequences and functional tRNA genes we used a comparative genomics approach using tRNA genes predicted for human, chimpanzee, mouse, rat, horse, dog, chicken, and fugu genomes. The fugu genome in particular, provides a good reference as it lacks many of the repetitive elements present in other vertebrate genomes [24]. While a number of functional tRNA genes may be filtered out due to the accumulation of mutations in neutral sites, we wanted to define a confident set of cow tRNA genes. With a 95% similarity threshold we identified 406 different tRNA genes with 41 distinct anticodons, encoding 135 tRNAs with unique sequences. However, this set of 405 tRNA genes was only capable of reading 55 of the 61 codons. We manually added the respective tRNA genes for the missing codons, which included 36 additional sequences. Many of these excluded tRNA genes were longer than the average 73 bp, due to the presence of a variable arm in the tRNA and as such lowered the sequence similarity score to less than 95%. We performed a cluster analysis of these cow tRNA genes and explored the relationships between the tRNA genes to understand more about tRNA gene evolution in the cow. From our analysis, tRNA genes with the same anticodon generally formed single clades with high posterior probability support. One exception that we investigated in more detail was tRNA genes from the glycine family. The vertebrates contain three distinct but related families of tRNA-Gly genes, with TCC, GCC and CCC anticodons. However, in mammals an additional family of tRNA-Gly^CCC ^genes is present (subfamily 2). This observed tRNA-Gly^CCC ^appears to have arisen in the ancestor of the eutherian mammals and marsupials by a mutation in the anticodon of a member of the tRNA-Gly^GCC ^family, as this new family was not identified among the non-mammalian genomes we investigated (Additional file 10). For the other more conserved subfamily 1 tRNA-Gly^CCC ^genes it is not clear whether the apparent absence of orthologs of this gene in the current platypus genome assembly is due to the incomplete nature of the assembly, or to loss of the genes in platypus, similarly for the identification of only one member of the family in the cow genome. Due to the redundancy of function, and the distinctive sequence differences between the two tRNA-Gly^CCC ^families it will be interesting to uncover the expression patterns of tRNA genes from the glycine family.

While we were able to gain an overview of tRNA genes within the cow genome, we wanted to trace the evolutionary history of cow tRNA genes across various organisms. We used phylogenetic profiles as a method to describe the conservation patterns of cow tRNA genes across 20 species. Generally, genomes that were phylogenetically more related to the cow genome contained a higher number of tRNA orthologs. However there was a large distinction in the number of tRNA orthologs (defined as sequences with ≥ 95% sequence identity) between vertebrate and invertebrate genomes. The number of cow tRNA orthologs among vertebrates is 2–3 folds larger than the number among invertebrates. Expansion of vertebrate genomes resulting in emergence of paralogous copies of tRNA genes may explain the larger numbers of tRNA orthologs observed in vertebrates. This is in line with the observation that the number of tRNA genes in a genome is positively correlated to the genome size [25]. However, due to the large evolutionary distance between cow and invertebrates, tRNA orthologs in invertebrates may have been omitted due to the stringency of our similarity threshold. The phylogenetic profiles also revealed a dispersed distribution of cow tRNA orthologs. Whilst roughly half of the cow tRNA orthologs are highly conserved and present in all vertebrate genomes the other half display a much more random distribution. This observation is in agreement with the hypothesis of a core and peripheral set of tRNA genes [26]. The authors suggest that tRNA gene evolution may be a repetitive process, which would explain the distribution of cow tRNA genes observed in our phylogenetic profiles.

## Conclusion

We have identified a set of highly conserved tRNA genes among vertebrate genomes as part of our analysis of the *B. taurus *genome. Our analyses revealed that while most tRNA isoacceptors seem to have evolved from a common ancestor, some may have different evolutionary histories. Our additional analysis of the glycine tRNA genes revealed two distinct families of tRNA-Gly^CCC ^genes, one of which seems to have been formed via a point mutation in the anticodon of a member of the tRNA-Gly^GCC ^family just prior to the radiation of mammals. Finally, our phylogenetic profiles of cow tRNA genes shows a large core set of tRNA genes conserved among vertebrate genomes, which highlights the importance of vertebrate genome expansion in relation to tRNA gene sets in genomes.

## Methods

### Sources of sequences

Human (*Homo sapiens*), chimpanzee (*Pan troglodytes*), dog (*Canis familiaris*), mouse (*Mus musculus*), rat (*Rattus norvegicus*), chicken (*Gallus gallus*), and fugu (*Takifugu rubripes*) tRNA gene sequences predicted by tRNAscan-SE and their respective tRNAscan-SE output files were downloaded from the genomic tRNA database [27] on the 11^th ^January 2008. The cow (*B. taurus*) (August 2006), horse (*Equus caballus*, January 2007), opossum (*Monodelphis domestica*, January 2006), platypus (*Ornithorhynchus anatinus*, March 2007), green anole lizard (*Anolis carolinensis*, February 2007), medaka (*Oryzias latipes*, April 2006), stickleback (*Gasterosteus aculeatus*, February 2006), tetraodon (*Tetraodon nigroviridis*, February 2004), zebrafish (*Danio rerio*, July 2007) and honey bee (*Apis mellifera*, January 2005) genome assemblies and sea urchin (*Strongylocentrotus purpuratus*, September 2006) scaffolds were downloaded as unmasked fasta files from the UCSC genome browser [28,29] The lamprey (*Petromyzon marinus*, March 2007) contigs, Pacific sea squirt (*Ciona savignyi*, July 2005) reftigs and lancelet (*Branchiostoma floridae*, March 2006) scaffolds were downloaded as unmasked fasta files from the UCSC test genome browser [30].

### De novo prediction of tRNA genes and identification of tRNA-derived repetitive elements

To identify putative tRNA genes in our downloaded sequences, we used tRNAscan-SE v. 1.23 with settings calibrated for eukaryotic genomes [15]. Fasta files of predicted tRNA genes were generated from the tRNAscan-SE output using in-house Perl scripts. For our tRNA analysis of the cow genome, pseudogenes were excluded from further analyses. To avoid potential redundancy of cow genome sequence data, tRNA gene predictions located on unordered genomic contigs (chrUn) were analyzed separately. To remove repetitive elements in the initial set of cow tRNA gene predictions, RepeatMasker (version open-3.1.8) [18] was used running with the following parameters: "-e cross_match", "-slow" and "-species cow". Consensus profiles for genomic repeat elements were obtained from RepBase 12.12 [31].

### Identifying functional cow tRNA genes using comparative genomics

We compared putative cow tRNA genes to pre-computed and annotated sets of tRNA genes from the human, chimpanzee, horse, dog, rat, mouse, chicken, and fugu genomes using the BLASTn program (version 2.2.17) [32] running on default settings. We used a 95% sequence similarity threshold as a filter for functional cow tRNA genes. Intronic sequences as predicted by tRNAscan-SE were removed from all tRNA genes before measuring similarity. Sequence similarity was calculated across the full length gapped pairwise alignment from the BLAST output, where a gap opening or base substitution counted as a mismatch. Finally, we identified a set of cow tRNA genes that were conserved with ≥ 95% sequence identity across all 8 genomes.

### Determination of phylogenetic profiles for cow tRNA genes using sequence homology

Phylogenetic profiles describe the presence-absence of homologous genes in genomes [33]. We constructed phylogenetic profiles for our set of 31,868 putative cow tRNA genes across a wide spectrum of genomes that included the dog, opossum, platypus chimpanzee, fugu, chicken, human, rat, mouse, horse, lizard, medaka, stickleback, tetraodon, zebrafish, lamprey, sea squirt, lancelet, sea urchin and honey bee. We used the BLASTn program running on default settings to compare all cow putative tRNA genes predicted using tRNAscan-SE to all sets of putative tRNA genes predicted from the respective genomes using tRNA scan-SE. Intronic sequences were not removed, as these sites are phylogenetically informative. tRNA genes with 95% or higher sequence similarity (calculated as above) were considered as orthologs. The phylogenetic profile of each cow tRNA gene is represented by a string of binary numbers 21 bits long, which corresponds to the number of genomes. The presence of an ortholog in another genome is represented as a 1 and the absence as a 0. Each position of a string corresponds to a respective genome. The positioning of each genome in each phylogenetic profile was arranged by the phylogenetic distance of the respective genome from cow as defined by NCBI taxonomy [34,35] i.e. the last vector of the phylogenetic profile is the most divergent genome from cow.

### Analysing the distribution of cow tRNA gene families

We inferred cluster relationships of all cow tRNA genes with orthologs in human, chimpanzee, horse, dog, mouse, rat, chicken and fugu. We aligned all unique tRNA sequences using T-Coffee [36] running on default parameters. Bayesian trees were constructed using MrBayes software v. 3.1.2 [37]. We used the recommended settings for Bayesian phylogenetic analysis of tRNA sequences, using a gamma distribution with four rate categories [38]. The General Time Reversible (GTR) model [39] was used for 1,000,000 Markov Chain Monte Carlo (MCMC) runs with a sampling frequency of 10,000. A burnin corresponding to 25% of the samples was used. Posterior probabilities (PP) values were assigned to every possible grouping of taxa in proportion to the number of times it is sampled. The number of times a tree is sampled is dependent on the likelihood of the tree, thus the PP value of a particular arrangement can be an estimate of confidence in that arrangement. The phylogenetic tree files were visualized and prepared using TreeView [40].

## Authors' contributions

DTPT performed detailed data analysis and wrote the manuscript. SM and WCB assisted with data analysis. BPD initiated this study and participated in its design, and the preparation of the final version of the manuscript. EAG coordinated this study, assisted with data analyses and drafted the final version of the manuscript. All authors have read and approved the final manuscript.

## Supplementary Material

Additional file 1 **Figure S1 – Bioinformatics pipeline for prediction of functional cow tRNA genes**. The pipeline shows the various stages of tRNA filtering for the cow genome using bioinformatics and comparative genomics. Each process is indicated by a rectangle and each data store by a slanted rectangle. For more details for each process please refer to the Methods section. Click here for file

Additional file 2 **Table S1 – Codon usage in *Bos taurus *genome compared against tRNA gene number**. The number of cow tRNA genes was predicted using our comparative approach (see methods). Codons that have been highlighted in bold are generally decoded by a single tRNA species due to wobble. Codon frequencies were obtained from . Click here for file

Additional file 3 **btau3.1_tRNA.fa – Fasta file of our confident set of cow tRNA genes**. The fasta file is annotated in the same format as seen in [26]. Click here for file

Additional file 4 **Figure S5 – Cluster relationships of cow serine tRNA genes**. Relationships between cow serine tRNA genes and a histidine tRNA gene, which is used as an outgroup. Posterior probability scores are shown for each cluster to support the strength of the respective clusters. Click here for file

Additional file 5 **Figure S6 – Cluster relationships of cow valine tRNA genes**. Relationships between cow valine tRNA genes and a histidine tRNA gene, which is used as an outgroup. Posterior probability scores are shown for each cluster to support the strength of the respective clusters. Click here for file

Additional file 6 **Figure S2 – Multiple sequence alignment of predicted cow glycine tRNA genes**. Multiple sequence alignment of all glycine tRNA genes with 20 bp of flanking sequence identified in the cow genome. BoxA (corresponding to nt 8–19) and BoxB (corresponding to nt 52–62) sites constitute internal promoter sites for RNA polymerase III. Conserved nucleotides are highlighted and indicated by asterisks. Click here for file

Additional file 7 **Table S2 – Summary of BLASTn matches and number of tRNA gene matches at a 95% similarity threshold**. Cow tRNA genes were blasted against the full set of tRNA genes from a respective genome. BLAST results were parsed using two different thresholds, an e-value of 0.0001 and using our comparative approach. Click here for file

Additional file 8 **Table S3 – Summary of most abundant phylogenetic profiles and the bovine tRNA genes constituting the profile**. The number of cow tRNA genes that make up the most abundant phylogenetic profiles are shown. Cow tRNA genes are grouped according to their amino acid specificity and anticodon. Click here for file

Additional file 9 **Figure S3 – Predicted secondary structure of the consensus Bov-tA2 sequence (downloaded from RepBase)**. Repetitive elements derived from tRNA maintain sequence similarity as well as structural features. Here we observe the similar stem loop structure of Bov-tA2 to tRNAs. Prediction of the secondary structure was done using tools available at Genomic tRNA database website [26]. Click here for file

Additional file 10 **Figure S4 – Phylogenetic relationships of human, chimpanzee, rat, mouse, dog, horse, cow, opossum, platypus, chicken, lizard, tetraodon, fugu, stickleback, medaka, zebrafish, lamprey, lancelet, sea squirt and sea urchin**. Phylogenetic relationships of many vertebrate genomes based on NCBI taxonomy. Click here for file
