# Supplementary figures and images for: Analysis of the complement and molecular evolution of tRNA genes in cow
Source: BMC Genomics. 2009 Apr 24;10:188. doi: 10.1186/1471-2164-10-188 (PMC2680898; doi:10.1186/1471-2164-10-188)

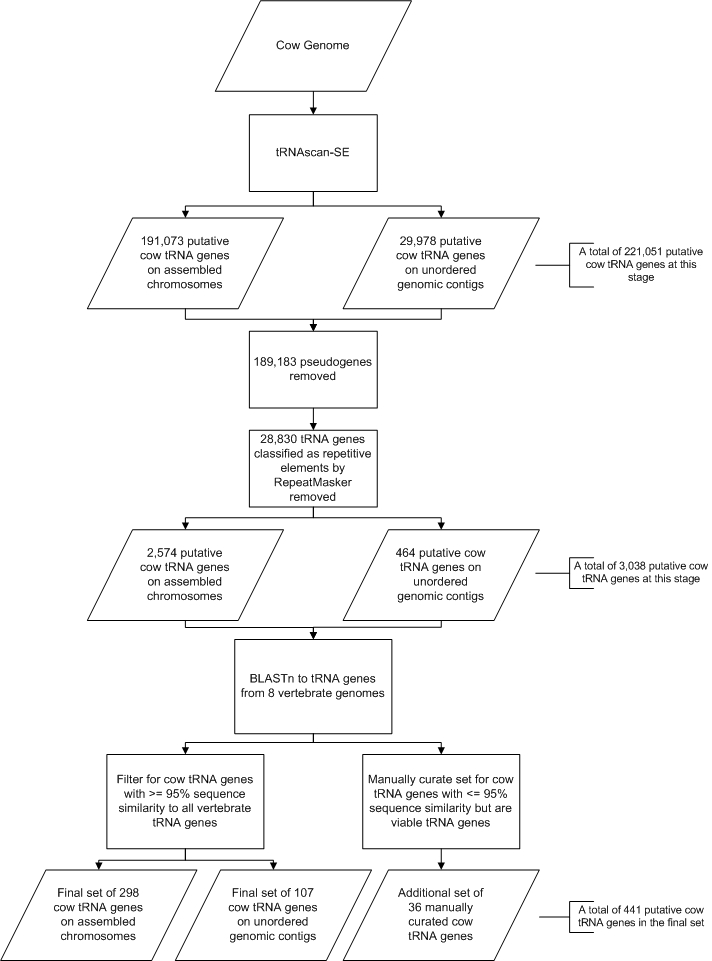

Supplement: Additional file 1 — Figure S1 – Bioinformatics pipeline for prediction of functional cow tRNA genes. The pipeline shows the various stages of tRNA filtering for the cow genome using bioinformatics and comparative genomics. Each process is indicated by a rectangle and each data store by a slanted rectangle. For more details for each process please refer to the Methods section. [file 1471-2164-10-188-S1.jpeg]

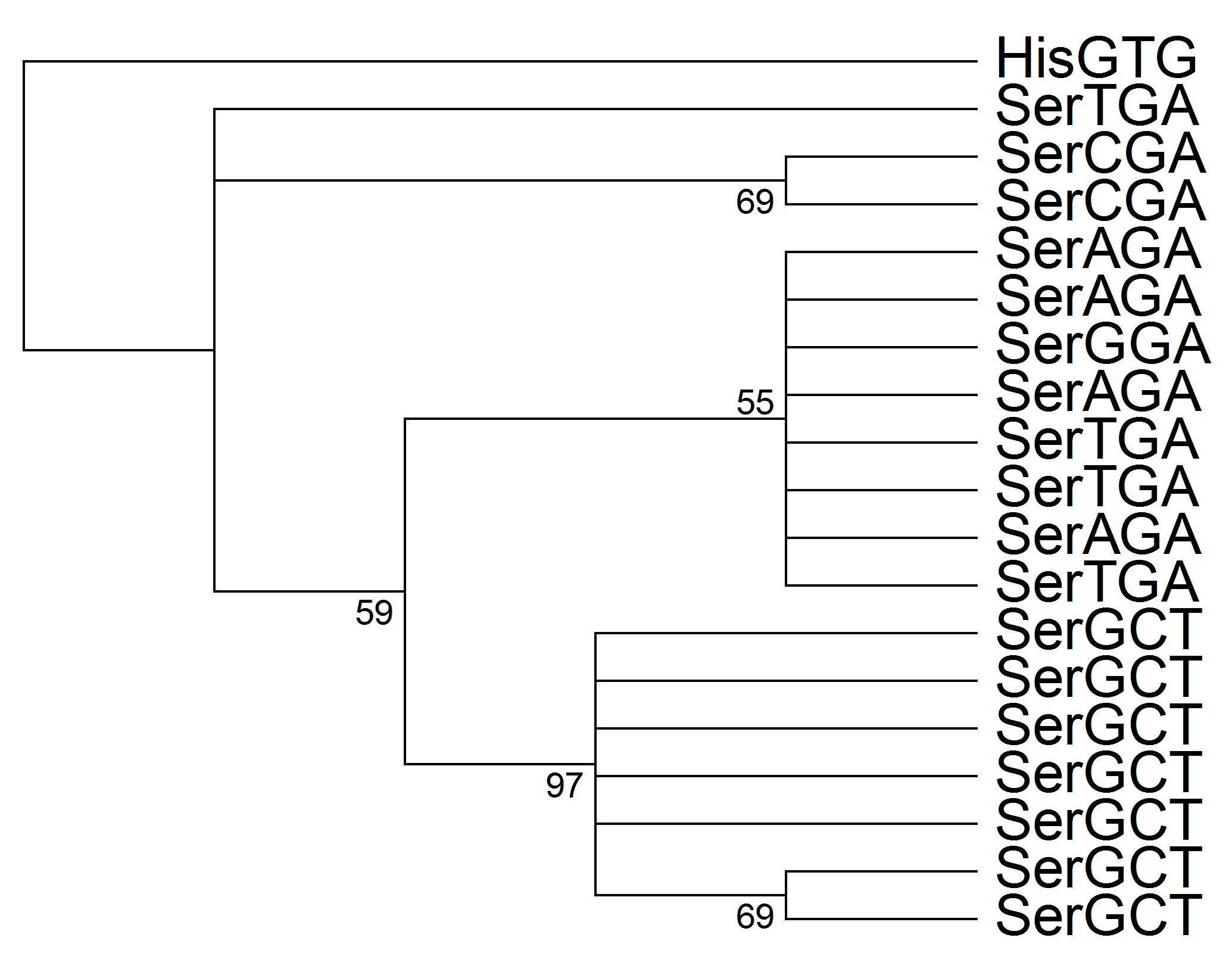

Supplement: Additional file 4 — Figure S5 – Cluster relationships of cow serine tRNA genes. Relationships between cow serine tRNA genes and a histidine tRNA gene, which is used as an outgroup. Posterior probability scores are shown for each cluster to support the strength of the respective clusters. [file 1471-2164-10-188-S4.tiff]

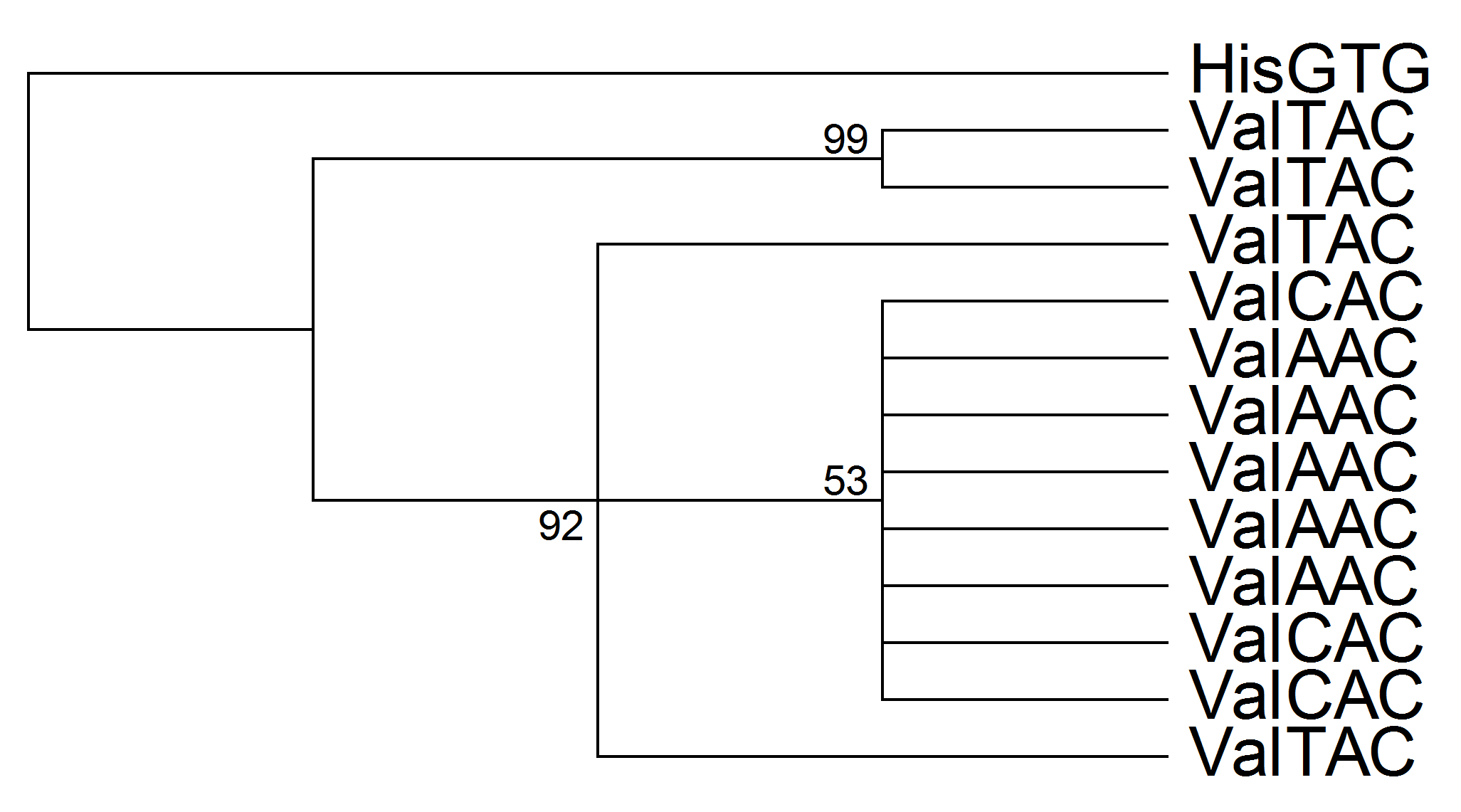

Supplement: Additional file 5 — Figure S6 – Cluster relationships of cow valine tRNA genes. Relationships between cow valine tRNA genes and a histidine tRNA gene, which is used as an outgroup. Posterior probability scores are shown for each cluster to support the strength of the respective clusters. [file 1471-2164-10-188-S5.tiff]

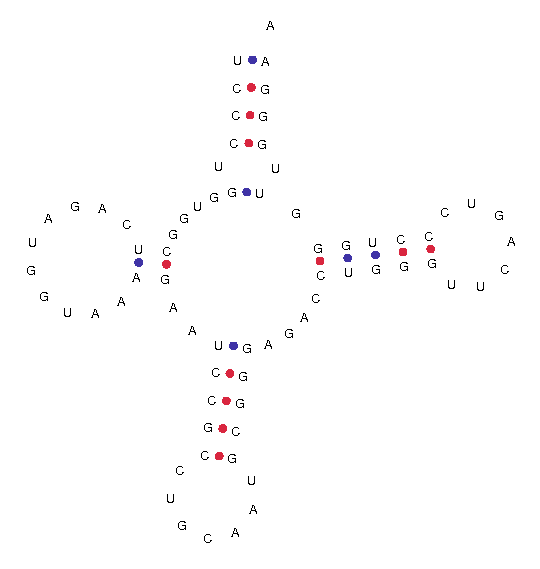

Supplement: Additional file 9 — Figure S3 – Predicted secondary structure of the consensus Bov-tA2 sequence (downloaded from RepBase). Repetitive elements derived from tRNA maintain sequence similarity as well as structural features. Here we observe the similar stem loop structure of Bov-tA2 to tRNAs. Prediction of the secondary structure was done using tools available at Genomic tRNA database website [26]. [file 1471-2164-10-188-S9.tiff]

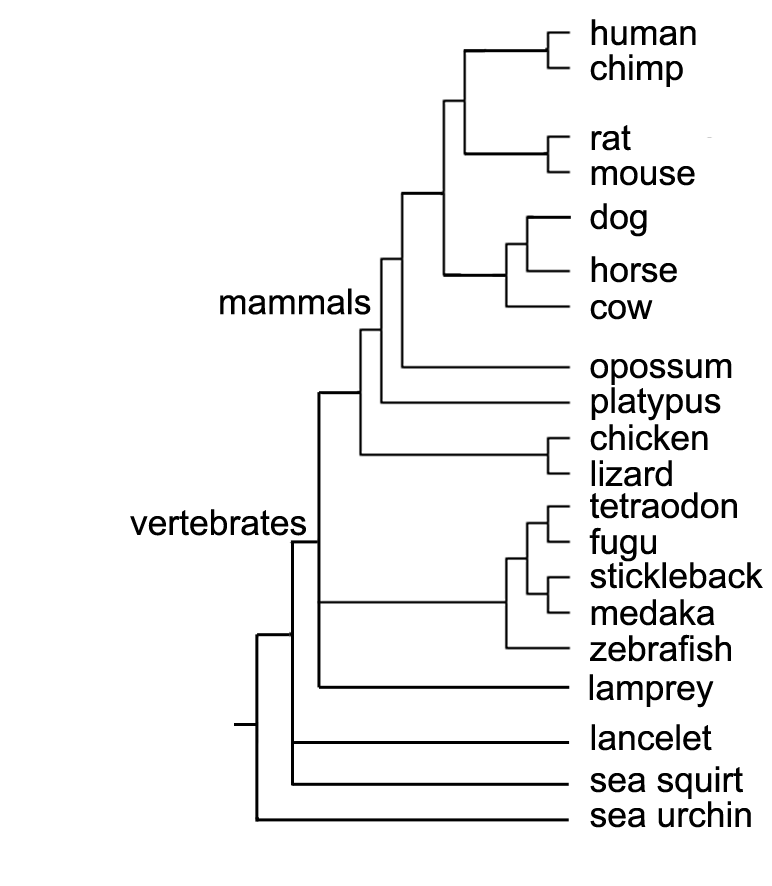

Supplement: Additional file 10 — Figure S4 – Phylogenetic relationships of human, chimpanzee, rat, mouse, dog, horse, cow, opossum, platypus, chicken, lizard, tetraodon, fugu, stickleback, medaka, zebrafish, lamprey, lancelet, sea squirt and sea urchin. Phylogenetic relationships of many vertebrate genomes based on NCBI taxonomy. [file 1471-2164-10-188-S10.tiff]
