# Supplementary material for: Analysis of the complement and molecular evolution of tRNA genes in cow
Source: BMC Genomics. 2009 Apr 24;10:188. doi: 10.1186/1471-2164-10-188 (PMC2680898; doi:10.1186/1471-2164-10-188)
Supplement: Additional file 6 — Figure S2 – Multiple sequence alignment of predicted cow glycine tRNA genes. Multiple sequence alignment of all glycine tRNA genes with 20 bp of flanking sequence identified in the cow genome. BoxA (corresponding to nt 8–19) and BoxB (corresponding to nt 52–62) sites constitute internal promoter sites for RNA polymerase III. Conserved nucleotides are highlighted and indicated by asterisks. [file 1471-2164-10-188-S6.pdf]

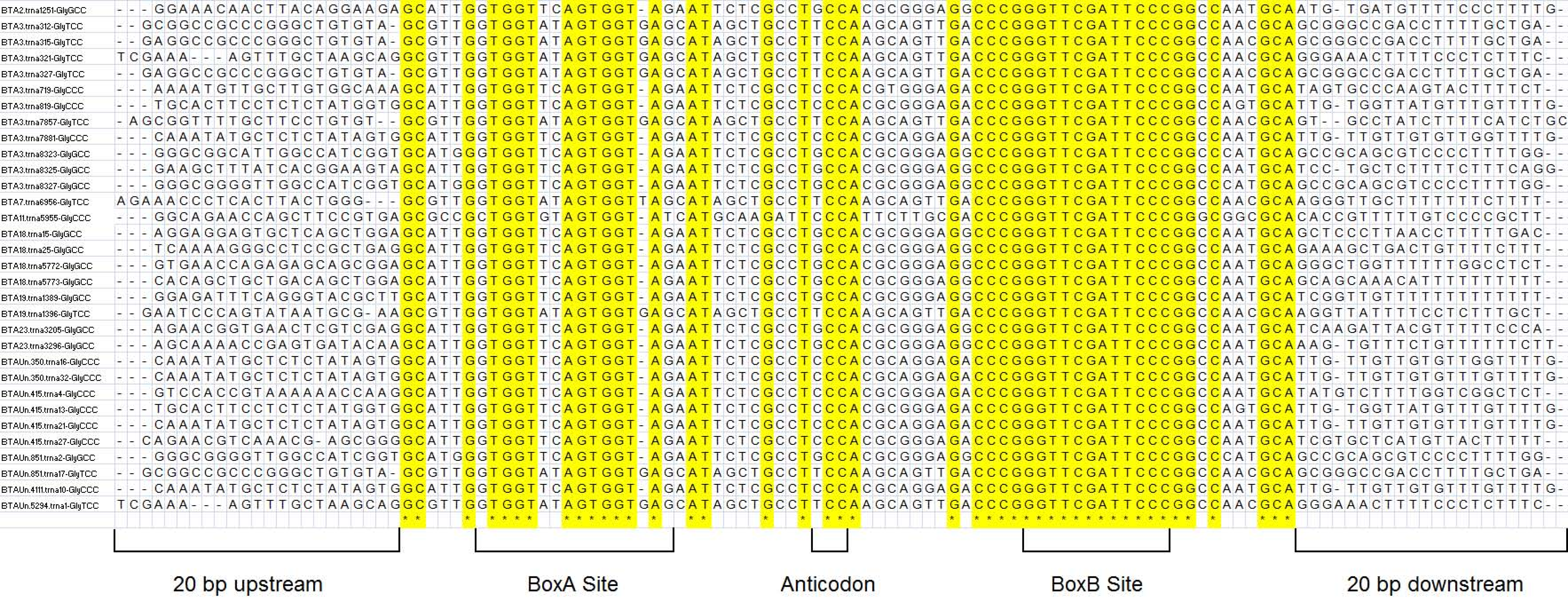
20 bp upstream
BoxA Site
Anticodon
BoxB Site
20 bp downstream
